# Immobilization and Stabilization of Enzyme in Biomineralized Calcium Carbonate Microspheres

**DOI:** 10.3389/fbioe.2020.553591

**Published:** 2020-10-09

**Authors:** Chan Hee Lee, Eon Seon Jin, Jin Hyung Lee, Ee Taek Hwang

**Affiliations:** ^1^Department of Life Science, Research Institute for Nature Sciences, Hanyang University, Seoul, South Korea; ^2^Center for Convergence Bioceramic Materials, Korea Institute of Ceramic Engineering and Technology, Cheongju-si, South Korea; ^3^Department of Food Biotechnology, Dong-A University, Busan, South Korea

**Keywords:** enzyme immobilization, enzyme stabilization, biomineralization, calcium carbonate, cross-linking

## Abstract

Biomineralized uniform and well-organized calcium carbonate microspheres were synthesized for enzyme immobilization, and the immobilized enzyme was successfully stabilized. The physicochemical parameters of calcium carbonate were studied using scanning electron microscopy with energy-dispersive X-ray spectroscopy, particle size analysis, X-ray diffraction analysis, Fourier-transform infrared spectroscopy, and surface area measurement. Additionally, Barrett-Joyner-Halenda adsorption/desorption analysis showed that the calcium carbonate microspheres provided efficient mesopore space for enzyme loading. As a model enzyme, carboxyl esterase (CE) was entrapped and then cross-linked to form an enzyme structure. In this aggregate, the cross-linked enzymes cannot leach out from mesopores, resulting in enzyme stability. The hydrolytic activities of the free and cross-linked enzymes were analyzed over broad temperature and pH ranges. The cross-linked enzyme displayed better activity than the free enzyme. Furthermore, the immobilized CE was found to be stable for more than 30 days, preserving 60% of its initial activity even after being reused more than 10 times. This report is expected to be the first demonstration of a stabilized cross-linked enzyme system in calcium carbonate microspheres, which can be applied in enzyme catalyzed reactions involved in bioprocessing, bioremediation, and bioconversion.

## Introduction

Enzymes are proteins that catalyze chemical reactions, reducing the initial energy input, by increasing the reaction rate. Based on enzyme processes relevant to environment-friendly features used in chemical transformation, enzymes are widely utilized in various fields such as food, pharmaceuticals, biodiesel, and biofuels (Datta et al., 2013; Hwang and Gu, 2013; Jesionowski et al., 2014). Additionally, enzymes usually function under mild conditions such as ambient temperature and pressure. Therefore, industrial applications of enzymes are increasing (Schoemaker et al., 2003). In industrial processes, free enzymes are destabilized, and it is difficult to reuse them efficiently (Iyer and Ananthanarayan, 2008). Enzyme immobilization allows overcoming this drawback by improving enzyme reactivity and stability (Mateo et al., 2007; Hanefeld et al., 2009). Immobilization of an enzyme can prevent it from structural denaturation caused by the external environment; thus, enzyme activity can be maintained from various reaction conditions by preserving storage stability. Besides, the immobilized enzyme can be easily separated from the reaction solution, and it can be easily washed and reused after measuring the activity (Hwang et al., 2012a, 2013).

Enzyme immobilization methods include physical adsorption, ionic and covalent bonds, and various techniques such as binding, entrapment, encapsulation, and cross-linking. Enzymes can be immobilized on various organic and inorganic materials or carriers. Synthetic organic polymeric beads such as Sepabeads and Amberlite; a variety of biopolymers, mainly water-insoluble polysaccharides such as cellulose, starch, agarose, alginate, chitosan, and electrospun nanofibers; and polymeric membranes are widely used as supports for immobilizing enzymes (Cantone et al., 2013; Hwang and Gu, 2013; Zdarta et al., 2018; Rodriguez-Abetxuko et al., 2020). Inorganic materials such as silica, magnetic particles, inorganic oxides, clay, and graphene oxide have been used for enzyme immobilization (Wang et al., 2014; Zucca and Sanjust, 2014; Wu et al., 2015; Xia et al., 2020). Among these, mesoporous materials have been applied to accommodate enzymes and maintain their activity in the pore space, owing to their pore size, connectivity, and high stability (Wang and Caruso, 2004; Xu et al., 2019). However, simple adsorption or entrapment into the pore space leads to rapid release from the pores, and reduces stability (Hwang et al., 2012b; Li and Wang, 2013).

To prevent the release of enzymes from mesoporous materials and improve stability, the cross-linking method is used (Kim et al., 2007). Cross-linking can be achieved via physicochemical methods such as heat treatment, application of alkaline conditions, mechanical agitation, photo-oxidation treatment, and the use of chemical reagents with an enzyme catalyst (Heck et al., 2013). A simple chemical reagent, glutaraldehyde (GA), is most widely used in biocatalyst design, and is the most powerful cross-linker (Barbosa et al., 2014). It has the advantage of self-reaction. Owing to the successful application of enzyme-immobilizing carriers, mesoporous silica has been widely used. However, for preparation, the high cost of the process (heat and solvent), mechanical stability under base conditions, size control, use of toxic silica precursors, and aggregation during the drying step can be disadvantages for its simple application in enzyme immobilization studies. Contrary to the several weaknesses of mesoporous silica, the advantages of calcium carbonate make it a good alternative enzyme immobilization carrier (Hwang et al., 2012a; Binevski et al., 2019). It can be used as an adsorbent or template, and synthesized as spheres with various particle sizes (Gower, 2008; Hwang et al., 2013). Moreover, it is suitable for living organisms because it is natural, non-toxic, and stable (Fakhrullin and Minullina, 2009). Furthermore, calcium carbonate is cheap, eco-friendly, and easy to synthesize with a simple scale-up process, in addition to being stable even under basic conditions (Binevski et al., 2019). Calcium carbonate and calcium carbonate–modified materials have previously been used in enzyme studies as immobilizing carriers and delivery vehicles. For example, several attempts have been made to encapsulate enzymes such as superoxide dismutase, alkaline phosphatase, pyruvate kinase, and lactate dehydrogenase and characterize the encapsulated enzyme in terms of the enzyme loading and efficiency, specific activity, and change in activity based on the morphological changes of calcium carbonate (Donatan et al., 2016; Muderrisoglu et al., 2018; Binevski et al., 2019). Additionally, the release behavior from the calcium carbonate particles has also been investigated (Roth et al., 2018; Abalymov et al., 2020). However, these studies did not focus on the stabilized enzyme system in calcium carbonate particles, the most important compound in industrial enzymatic bioconversion. In pharmaceutical and fine chemical industries, bioconversion processes necessitate a specific reactor design and enzyme activity for effective execution of the enzymatic processes.

Therefore, as a model study, we investigated enzyme stabilization by immobilizing carboxyl esterase (CE) from *Rhizopus oryzae* into the pores of biomineral calcium carbonate microspheres. Biomineralized calcium carbonate can be synthesized as a well-formulated uniform carrier. First, Biomineralized calcium carbonate can be synthesized into well formulated uniform carrier; The prepared mesoporous spherical calcium carbonate microspheres with a size of 5.5 ± 1.8 μm were fully characterized using scanning electron microscopy (SEM). Energy-dispersive X-ray spectroscopy (EDS), particle size analyzer (PSA), X-ray diffraction (XRD), Fourier-transform infrared spectroscopy (FT-IR). Additionally, Brunauer-Emmett-Teller (BET) was used to analyze the surface area, pore volume, adsorption pore size, and desorption pore size. First, the CE was adsorbed inside of the prepared calcium carbonate and then cross-linked by treatment with GA to design stabilized enzyme structures. It was found that the activity was preserved for 30 days; the catalytic activity was stable even after 10 reuses, exhibiting high storage stability and recyclability. This study gives the applications of a stable and recyclable cross-linked enzyme in calcium carbonate microspheres and will contribute to practical enzyme-based process applications.

## Materials and Methods

### Materials

Calcium chloride (Sigma), ammonium carbonate (Sigma), sodium phosphate monobasic (Sigma), sodium phosphate dibasic (Sigma), *N,N*-dimethylformamide (DMF) (Sigma), and Tris–HCl (1 M, Bioneer, Alameda CA, United States) were purchased. The model enzyme CE from *Rhizopus oryzae* (Sigma) was used for immobilization, and the substrate *p*-nitrophenyl butyrate (Sigma) was purchased from Sigma-Aldrich. *p*-nitrophenol (Sigma) was used for the standard curve for products by enzymatic reaction. Finally, a BCA protein assay kit (Pierce, Rockford, IL, United States) for measuring enzyme loading was purchased from Sigma.

### Synthesis of Calcium Carbonate Microspheres

To synthesize biomineralized calcium carbonate microspheres, 200 mM of CaCl_2_ solution (distilled water:acetone = 5:1) was mixed for dissolution. (Na_4_)_2_CO_3_ solution (200 mM) was added to CaCl_2_ solution with vigorous stirring for 10 min. After synthesis, the sample was washed twice with ethanol to remove solution and then dried at 60°C in a dry oven. After drying, the powder samples were collected and stored at room temperature.

### Characterization of CaCO_3_ Microspheres

The morphological and elemental analysis of CaCO_3_ microspheres was performed using FE–SEM equipped with energy-dispersive X-ray spectroscopy (EDAX) (TESCAN, Czech Republic). Pt sputtered coating was done before SEM analysis. BET surface area and pore size were obtained using a Tristar II (Micromeritics, United States). Pore-size distributions and pore volume were calculated using the Barett–Joyner–Halenda equation. FT-IR analysis was performed using an FT-IR spectrometer (Jasco, United States) range of 650–4000 cm^–1^. XRD patterns were analyzed by a Rigaku Mini Flex 600 (Rigaku, Japan) using Cu Kα radiation (λ = 1.5418 Å) at scanning rate of 5.00°min^–1^. Particle size analysis was performed using laser scatter particle size distribution analyzer LA-960 (Horiba, Japan).

### Enzyme Immobilization

Immobilization of the enzyme into the calcium carbonate pores was performed *via* two-step process. First, 1.5 mL CE solution (0.66 mg/mL) was added to a glass vial containing 10 mg of calcium carbonate and vortex strongly for 30 s. After the sonication, the sample was incubated at 200 rpm for 30 min to be adsorbed into the calcium carbonate pores. To cross-link the adsorbed enzyme, 1.5 mL of 1% GA solution (diluted with 100 mM phosphate buffer, pH 8.0) was added to the enzyme and calcium carbonate mixture samples. The sample was stored at room temperature for 60 min without any movement, and then incubated for 60 min at 200 rpm to prepare cross-link between CE molecules. After that, 100 mM Tris–HCl (pH 8.0) was used to remove unreacted GA by centrifugation. The sample was consecutively washed thrice with buffer and stored in 100 mM phosphate buffer (pH 8.0) at 4°C until the next use. The amount of CE loaded into calcium carbonate was measured by BCA protein assay kit.

### Activity and Stability Measurement

The activity of cross-linked CE in biomineral calcium carbonate microspheres was determined using the concentration of *p*-nitrophenol resulting from hydrolysis of *p*-nitrophenyl butyrate substrate dissolved in *N,N*-dimethylformamide. For activity measurement, *p*-nitrophenyl butyrate substrate was dissolved in *N,N*-dimethylformamide to prepare 10 mM concentration, and then added to 100 mM phosphate buffer (pH 8.0) solution containing 0.042 mg of cross-linked CE in CaCO_3_ to be 0.5 mM of final substrate concentration. The enzyme reaction was performed by shaking for 12.5 min at 200 rpm with 2.5 min intervals during the reaction. At each interval time, absorbance was measured at 400 nm. The activity was calculated as the average of the slope values measured by absorbance per reaction time in proportion to the concentration of *p*-nitrophenol. After the measurement, the immobilized CE was washed five times with 100 mM phosphate buffer (pH 8.0) and stored at room temperature.

The effect of pH on the activity of free and immobilized CE was performed over pH range 6.0–10.0 and at room temperature. The effect of temperature on CE activity was carried out over 15–55°C, at pH 8.0. The activity was measured according to the above experimental protocol. The results for pH and temperature are presented in a normalized form with the highest activity of each set assigned by the value of 100% activity.

The storage and recycling stability were measured using the above experimental protocol, and the relative activity at each time point was calculated as the percentage ratio of residual activity at each time to the initial activity of each sample. All samples were measured thrice for standard deviation. The standard deviation is represented by error bars in the figure.

## Results and Discussion

### Characteristics of the CaCO_3_ Microspheres

**FIGURE 1:**
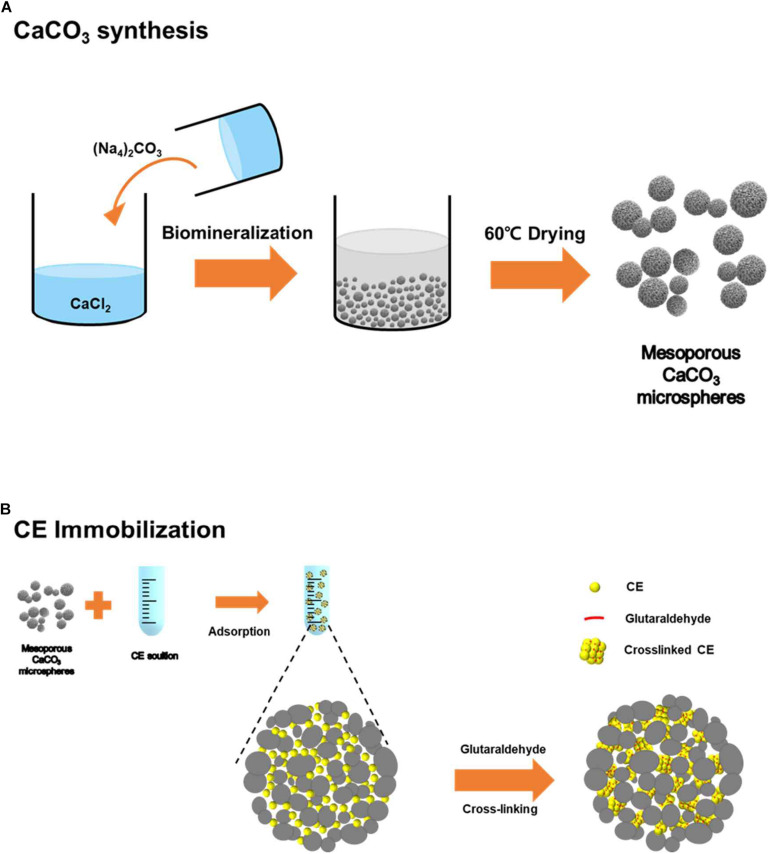
Schematic illustrations of **(A)** CaCO_3_ microsphere preparation and **(B)** enzyme immobilization.

**FIGURE 2:**
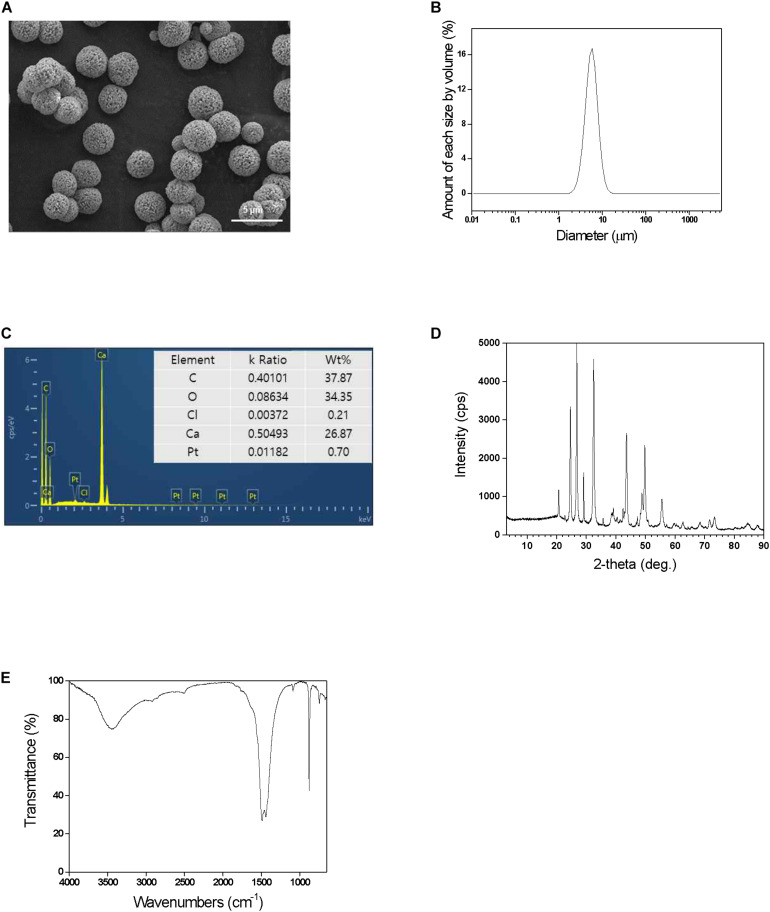
**(A)** SEM images, **(B)** particle size distribution, **(C)** energy-dispersive X-ray spectroscopy (EDS), **(D)** XRD patterns, and **(E)** FT-IR spectrum of the prepared CaCO_3_ microspheres.

The calcium carbonate microspheres were successfully synthesized based on the mineralization process as described in Figure 1A. The fabricated CaCO_3_ were successfully characterized using SEM with elemental analysis (EDS), PSA, FT-IR, XRD, BET, and BJH adsorption/desorption. When analyzed, Figure 2A shows the highly organized shape of calcium carbonate, as shown in the SEM image, well-dispersed spherical structure with the diameter of 4–6 μm. The particle size analysis obtained by PSA, showing 5.5 ± 1.8 μm size distribution (Figure 2B), and EDS was used to confirm CaCO_3_ surface (Figure 2C). The XRD patterns of particle confirmed that well-crystallized mixed vaterite and aragonite phase, with their characteristic diffraction peaks of 2θ values at about 25.4 (1 0 1), 37.9 (0 0 4), 48.2 (2 0 0), 54.0 (1 0 5), 55.1 (2 1 1), and 62.9 (2 0 4), indicating nature of the calcium carbonate, were obtained (Figure 2D). Additionally, FT-IR spectrum indicated the typical CaCO_3_ peak coincident with the broad band at 1394 cm^–1^ (υ3 asymmetric CO_3_^2–^) and with the sharp band at 871 cm^–1^ (υ2 asymmetric CO_3_^2–^) (Figure 2E). The spherical calcium carbonate with mesoporous property is the key step to enzymes loading into the structured materials for efficient enzyme immobilization. Therefore, the CaCO_3_ microspheres are also expected to have pore sizes and volume enough for spaces without limitation. In fact, porous structure was confirmed through BJH analysis using the representative N_2_ adsorption/desorption data, shown in Table 1A. The BET surface area of the hybrid particles was 6.88 m^2^ g^–1^, and the fraction of vacant mesopores provided pore volume of 0.048 m^2^/g, leading to entrap enzyme. In addition, average pore size from adsorption and average pore size from desorption of microspheres were 30.914 nm and 27.937 nm, respectively (Table 1A). These unique structures are a suitable mesoporous material for enzyme immobilization, which allows facile biocatalysis.

**TABLE 1:** Characteristics of **(A)** CaCO_3_ microspheres, and **(B)** free CE and cross-linked CE **(A)**.

(A)				

	*S*_BET_	*V*	*d*_ad_ (average)	*d*_de_ (average)
	(m^2^/g)	(cm^3^/g)	(nm)	(nm)
CaCO_3_ microspheres	6.888	0.048	30.914	27.937

**(B)**			

	**Specific activity**	***K*_m_**	***V*_max_**
**Sample**	**(mM min^–1^ mg^–1^)**	**(mM)**	**(mM min^–1^)**

Free CE	48.32	0.25 ± 0.02	2.82 ± 0.13
Cross-linked CE	7.63	0.94 ± 0.25	0.82 ± 0.16

**S*_*BET*_ is the BET surface area; *V* is the total pore volume; pore size, *d*_*ad*_ and *d*_*de*_ were calculated using the BJH (Barett–Joyner–Halenda) method.*

### Enzyme Immobilization

For enzyme immobilization, CE was prepared *via* a two-step process: enzyme adsorption and cross-linking into the prepared biomineralized calcium carbonate microspheres as described in Figure 1B. CE enzyme (1.0 mg) was dissolved in 1.5 mL buffer solution under 200 rpm shaking condition at room temperature. CE was adsorbed onto the exterior surface of the particles or in the inter-particle space. After washing with 100 mM sodium phosphate buffer (pH 8.0), enzyme adsorption was completed. To prevent the enzyme leaching in the calcium carbonate pores space, adsorbed enzyme molecules were cross-linked *via* GA chemical linker. The cross-linked CE improves loading quantity, loading efficiency, and prevents denaturation and release of enzymes, thus ensuring the stability of enzyme activity. The enzyme loading efficiency and loading amount are different depending on the concentration of enzyme and amount of calcium carbonate used as a carrier. Therefore, the experiment is optimized by keeping the amount of enzyme with 10 mg of calcium carbonate microspheres. For measurement enzyme loading, BCA protein assay kit was used. About 0.2 mg of CE was immobilized in 10 mg of calcium carbonate microsphere during adsorption and washing steps. Unfortunately, CE loading could not be easily measured because cross-linked insoluble form of CE aggregates and cannot be used for protein assay kit. For the comparative studies, we assumed that enzymes are well retained within the mesopores of calcium carbonate upon GA treatment during immobilization. This means that about 20% of the enzyme is loaded during the adsorption and additional GA cross-linking process in the calcium carbonate pores.

### Characteristics of Immobilized Enzyme

To characterize immobilized CE in the CaCO_3_ microspheres, we investigated CE catalyzed hydrolysis enzymatic reactions. The specific activities of free and cross-linked CE were 48.32 and 7.63 mM min^–1^ mg^–1^, respectively. The activity of free CE was 16% more than that of cross-linked CE (Table 1B), indicating that the flexibility of the enzyme may reduce low diffusion rate of the reactants and products, resulting in low activity values. Most enzymes immobilized using traditional methods displayed <10% of specific activity retention (Zhang et al., 2011); therefore, 16% represents a high retention of specific activity along with maintaining this activity and preserving stability, thus preventing the denaturation and autolysis of CE (Table 1B).

**FIGURE 3:**
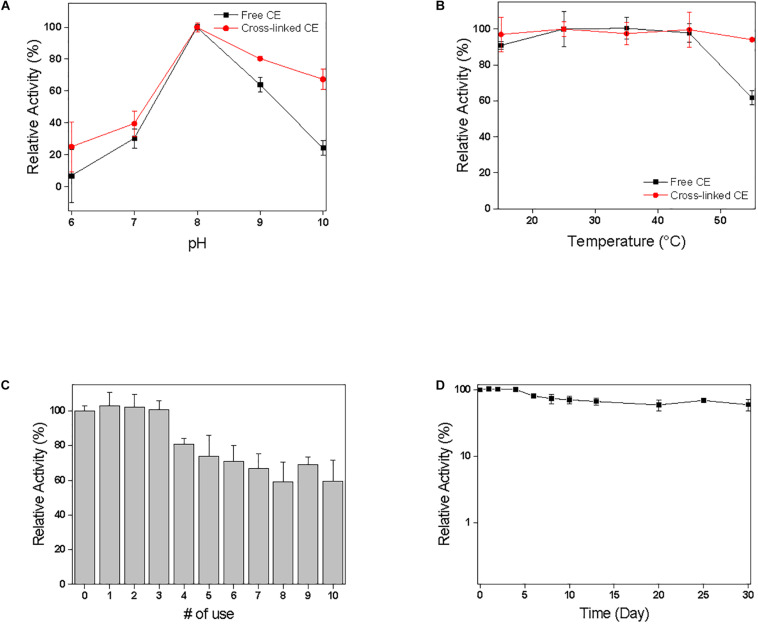
Effect of **(A)** pH and **(B)** temperature on enzyme activity in comparison of free CE and cross-linked CE. The results were normalized with the highest value of each set being assigned the value of 100% activity. **(C)** Recycling and **(D)** Storages stability of cross-linked CE. The relative activity was calculated based on the initial activity value of 100%.

To investigate efficiency of immobilization, the stability of free and cross-linked CE at different pH and temperature conditions was examined. The effect of pH effect on the activity was examined in the range of 6.0–10.0 at 25°C, and the highest CE activity was observed at pH 8.0. During the pH study, immobilized CE was more stable compared to free CE, indicating that enzyme activity is preserved after immobilization (Figure 3A). These results could probably be attributed to the stabilization of enzymes resulting from its multipoint attachments on the surface of CE *via* GA treatment. Furthermore, the microenvironment of the immobilized enzyme in inorganic CaCO_3_ microspheres might lead to the displacements in the pH activity profile arising from electrostatic interactions with the negative charged hydrophobic CaCO_3_ surfaces (Vescovi et al., 2016). During temperature profiling in the temperature range of 15–55°C, at pH 8.0, compared to free CE, immobilized CE was found to be more stable. At 55°C, the activity of free CE reduced by 62%, whereas cross-linked CE exhibited 94% activity (Figure 3B). It shows that immobilized CE could withstand higher temperature conditions compared to free CE and temperature profile of immobilized CE is broader than free CE activity at all temperatures. It was confirmed that the abnormality was maintained, and the immobilization resulted in an increase in the resistance of the CE to pH and temperature. All range of pH and temperature, the activity of immobilized CE was higher than that of free CE (Figure 3), indicating that enzyme activity is preserved after immobilization. The resistance to temperature and pH was increased due to the effect of cross-linking immobilization strategy in the pores of the calcium carbonate microspheres.

### Storage and Recycling Stability of Immobilized Enzyme

The stability of immobilized CE was checked by measuring time-dependent absorbance change in *p*-nitrophenol production by CE catalyzed reaction when each sample was incubated in 100 mM sodium phosphate buffer (pH 8.0) under shaking at 200 rpm (Figures 3C,D). In our previous study, free CE activity retained <1% residual activity within a week. Figures 3C,D shows the results of the reuse and storage stability of the immobilized enzyme in the pores of biomineralized calcium carbonate microspheres. The relative activity of the cross-linked CE is defined as a ratio to the initial activity value; therefore, the initial activity value is assumed to be 100%. To investigate the reuse and storage stability of the enzyme, after measurement of enzyme activity, the immobilized enzyme was washed five times with 100 mM phosphate buffer (pH 8.0) and stored at room temperature. As shown in Figures 3C, after reusing the enzyme 10 times, >60% of the activity was maintained depending upon the initial activity value. Therefore, cross-linked CE was able to preserve high activity and stability compared to the initial activity value even after repeated use. In addition, during the storage period of 30 days (Figure 3D), immobilized CE maintained high activity relative to the initial activity value and maintained stability. The high stability of cross-linked CE can be preserved because cross-linking prevents the release of enzymes, which are adsorbed into the pores of calcium carbonate, and inhibits the denaturation of the enzyme structure by inducing multiple-point chemical linkages. The enzyme stabilization attained can be explained by the “ship-in-a-bottle” mechanism. The cross-linking of the enzyme prevents its leaching through the smaller bottleneck pore structure of the mesoporous calcium carbonate particles. The success with highly stable immobilized enzyme in the CaCO_3_ has opened up a great potential for a variety of enzymatic synthetic reactions to be practically realized based on this biomineral based process design.

## Conclusion

Biomineral calcium carbonate was fabricated and well characterized using XRD, TEM, SEM, EDS, and BET surface area, pore size, and volume of adsorption and desorption pores. CE from *Rhizopus oryzae* was successfully immobilized by adsorption, followed by cross-linking in the biomineral calcium carbonate pores. During immobilization, 20% of the CE enzyme was loaded in the pores of calcium carbonate, and 16% of specific activity was maintained compare to free CE. The enzyme retained >60% of relative enzyme activity even after 10 reuses, when stored for 30 days at room temperature, indicating high recycling and storage stability. Therefore, this method, which successfully used natural calcium carbonate to immobilize enzyme and confirmed its stability as a biological resource, showed the possibility of applying another enzyme. It can be used as a new functional material.

## Data Availability Statement

All datasets presented in this study are included in the article/supplementary material.

## Author Contributions

EH and JL contributed to conception and design of the study. CL performed the experiments and analyzed the data. EJ wrote sections of the manuscript. CL wrote the first draft of the manuscript. EH and JL finalized the manuscript. All authors contributed to manuscript revision and read and approved the submitted version.

## Conflict of Interest

The authors declare that the research was conducted in the absence of any commercial or financial relationships that could be construed as a potential conflict of interest.
